# Genetic characterization of co-trimoxazole non-susceptible *Streptococcus pneumoniae* isolates from Indonesia

**DOI:** 10.1099/acmi.0.000271

**Published:** 2021-10-25

**Authors:** Dodi Safari, Hanifah Fajri Maharani Putri, Arrayan Bimantari, Wisiva Tofriska Paramaiswari, Wisnu Tafroji, Miftahuddin Majid Khoeri, Korrie Salsabila

**Affiliations:** ^1^​ Eijkman Institute for Molecular Biology, Jakarta, Indonesia; ^2^​ Biology Program, Surya University, Tangerang, Indonesia; ^3^​ Faculty of Biology, Gajah Mada University, Jogjakarta, Indonesia

**Keywords:** co-trimoxazole, dihydrofolate reductase, dihydropteroate synthase, *Streptocococcus pneumoniae*

## Abstract

We investigated the genetic variation of *folA* and *folP* genes encoding dihydropteroate synthase (DHPS) and dihydrofolate reductase (DHFR) enzymes amongst trimethoprim/sulfamethoxazole (co-trimoxazole) non-susceptible *Streptococcus pneumoniae* isolated from the Indonesian population. Archived *S. pneumoniae* isolates were screened for the presence and analysis of *folA* and *folP* genes using the polymerase chain reaction sequencing method. We found that 80 % of co-trimoxazole non-susceptible isolates (*n*=30/39) showed a 6 bp insertion in the sulphonamide-binding site of DHPS. The Asp-92-Ala and Ile-100-Leu substitutions were more common on DHFR (42 %; 22/53) followed by the Asp-92–Ala, Glu-94–Asp and Ile-100–Leu substitutions (32 %; 17/53). The combination of the Ile-100–Leu substitution at the DHFR region and the 6 bp insertion was the most dominant combination among isolates having both *folA* and *folP* genes.

## Introduction

Trimethoprim/sulfamethoxazole (co-trimoxazole) is an inexpensive and broad-spectrum antimicrobial drug that is still widely used in developing countries [1]. It is commonly administered as a prophylactic to protect against opportunistic infections for HIV-infected individuals and as a priority intervention for HIV-infected pregnant women [2]. It has been suggested that co-trimoxazole increased the risk of carrying pneumococci slightly among HIV-infected children in Zambia [3]. It has been used as a treatment option for a range of pneumococcal diseases, particularly in children [4]. In Indonesia, this drug was reported to be the second most common antibiotic used after the penicillin class to treat gastrointestinal disorders, respiratory system disorders, unspecified pyrexia, metabolism and nutrition disorders [5–7].

Resistance to co-trimoxazole among pneumococcal diseases has increased worldwide [4]. In Indonesia, the percentage of co-trimoxazole non-susceptible *Streptococcus pneumoniae* has increased over time. In 1997, it was reported that 12 % of *S. pneumoniae* isolated from 484 healthy children (0–25 months of age) in Lombok, Indonesia were non-susceptible to sulfamethoxazole [8]. Furthermore, a pneumococcal carriage study conducted in Lombok also reported that the percentage of co-trimoxazole non-susceptible *S. pneumoniae* increased to 62 % in 2012 [9]. Resistance to co-trimoxazole is associated with mutation on the *folP* and *folA* genes encoding dihydropteroate synthase (DHPS) and dihydrofolate reductase (DHFR) enzymes, respectively. Genetic variation of these genes plays an important role in co-trimoxazole resistance [10]. The polymorphisms of these genes have also been reported to have an association with the level of resistance [11] In this study, we aimed to analyse and characterize the genetic variations of *folA* and *folP* genes correlated with co-trimoxazole resistance among *S. pneumoniae* isolates in Indonesia.

## Methods

Seventy-eight archived isolates of *S. pneumoniae* obtained from nasopharyngeal swab specimens from the Indonesian population were used in this study. The *S. pneumoniae* strains were isolated from nasopharyngeal swab specimens from Indonesia [9] . In this study, we included different pneumococcal serotypes, i.e. 23F (*n*=18), 6A/B (*n*=16), 19F (*n*=10), 14 (*n*=9), 15B/C (*n*=4), non-typeable (NT; *n*=4), 31 (*n*=2), 3 (*n*=1), sg18 (*n*=2), 20 (*n*=1), 34 (*n*=1), 38 (*n*=1), 23F (*n*=1), 17F (*n*=1), 22F (*n*=1) and 35A/C/42 (*n*=1). The co-trimoxazole minimal inhibitory concentration (MIC) was measured using ETEST strips (MIC range: 0.002–32 µg ml^−1^) (bioMérieux SA, Marcy l’Etoile, France) according to the manufacturer’s instructions.

The polymerase chain reaction (PCR) and DNA sequencing targeting genes for *folA* and *folP* genes were performed as previously described [10]. The isolates were subcultured onto a 5 % sheep blood agar plate and incubated at 37 °C with 5 % CO_2_ for 20 h. Fresh culture was harvested into 300 µl TE buffer in a 1.5 ml microcentrifuge tube and vortexed. The bacterial suspension was heated at 100 °C for 5 min and then immediately placed in −20 °C conditions for 5 min before being centrifuged at 13 000 ***g*** for 10 min [12, 13]. The PCR reaction mixture comprised GoTaq Green Master Mix (Promega, Madison, WI, USA), the primers for the *folP* gene: *folP* forward 5′-GTCAAGTAAAGCCAATCATG-3′ and *folP* reverse 5′-AATTTTCCGCTTCATCAGC −3′ and the primers for the *folA* gene: *folA* forward 5′-TGTAAGCTATTCCAAACCAG-3′ and *folA* reverse 5′-CTACGTTCCATTAGACTTCC-3′ at 10 µM concentration, 1.0 µL of DNA template and nuclease-free water to a final volume of 50 µl. The PCR condition for *folP* gene was set as follows: 95 °C for 5 min followed by 35 cycles of 94 °C for 60 s, 51 °C for 60 s and 72 °C for 90 s, with a final extension at 72 °C for 7 min. The PCR condition for *folA* gene was set as follows: 95 °C for 5 min followed by 35 cycles of 94 °C for 60 s, 53 °C for 60 s and 72 °C for 30 s, with a final extension at 72 °C for 7 min. The amplicons for *folP* and *folA* genes were visualized with gel electrophoresis for approximately 600 and 900 bp, respectively. The amplicons were sequenced using the BigDye Terminator v3.1 labelled cycle sequencing kit (Applied Biosystems, USA) according to the manufacturer’s instructions. DNA sequences of *folP* (*n*=32) and *folA* (*n*=51) genes were submitted to the GenBank database with accession numbers MW816655–6685, MW816687–6694, MW816696–6707, MW835933–5961 and MW835963–5965.

## Results

In this study, co-trimoxazole non-susceptible isolates comprised 78 % (61/78) of the archived *S. pneumoniae* isolates. The MIC range for co-trimoxazole for non-susceptible isolates was 0.75/14.25 to >32/608 µg ml^−1^. Eighty-nine per cent of non-susceptible isolates (54/61) were vaccine-type strains. The other isolates (17/78) were susceptible to co-trimoxazole (MIC value <0.5/9.5 µg ml^−1^). We managed to sequence 55 isolates out of 78 isolates, while others (29 %; 23/78) were not sequenced as *folP* was not amplified. Among these 55 sequenced isolates, we identified that 71 % (39/55) of isolates were non-susceptible to co-trimoxazole and 29 % (16/55) of the isolates were susceptible to co-trimoxazole. The co-trimoxazole non-susceptible isolates had an insertion of 6 bp in *folP* at a higher level than susceptible isolates, i.e. non-susceptible isolates=77 % (30/39) vs susceptible isolates=44 % (7/16). We also found that 2 different types of 3 bp insertion and 11 different types of 6 bp insertion were observed in the *folP* gene (Table 1). The most prevalent 6 bp insertion among non-susceptible and susceptible isolates was ST*RP*RPGSSYVEIE [54 % (21/39) and 19 %(3/16), respectively] (Table 1). In this study, we observed that the MIC values for isolates with the 6 bp insertion (MIC ranged from 0.064/1.216–32/608 µg ml^−1^) were higher than those for the isolates with the 3 bp insertion (MIC ranged from 0.38/7.22–16/304 µg ml^−1^), and the wild-type isolates (MIC ranged from 0064/1.216–6/114 µg ml^−1^).

**Table 1.** Insertion of the *folP* gene encoding the DHPS enzyme in *S. pneumoniae* isolates from the Indonesian population

*FolP* insertion	DHPS variation*	No. (*n*=55)	Non-susceptible isolates (*n*=39)*, *n* (%)	Susceptible isolates (*n*=16)†, *n* (%)
3 bp	STRPG*R*SSYVEIE	5	3 (8)	2 (13)
	STRPGSS*C*YVEIE	2	2 (5)	0
6 bp	STRPG*SS*SSYVEIE	3	2 (5)	1 (6)
	STRPGSS*YG*YVEIE	1	0	1 (6)
	STRPGSSYV*EI*EIE	1	0	1 (6)
	STRPGSS*YV*YVEIE	2	2 (5)	0
	ST*RP*RPGSIYVEIK	1	1 (3)	0
	ST*RP*RPGSSYDEIE	1	1 (3)	0
	ST*RP*RPGSSYIEIE	1	1 (3)	0
	ST*RP*RPGSSYVEIE	24	21 (54)	3 (19)
	ST*RP*RPGSSYVEIV	1	0	1 (6)
	ST*RP*RPVSSYVEIK	1	1 (3)	0
	ST*RS*RAGSSYVEIE	1	1 (3)	0
No mutation	STRPGSSYVEIE	11	4 (10)	7 (44)

*Non-susceptible isolates with MIC range 075–32 µg ml^−1^.

†Susceptible isolates with MIC range <0.75 µg ml^−1^.

Among 78 isolates, we managed to obtain *folA* sequences from 69 isolates while the other 9 isolates were not processed for sequencing, since the *folA* could not be generated using the primers employed in this study. Among these 69 isolates, we defined 77 % (53/69) isolates as non-susceptible, while the other 23 % (16/69) were defined as susceptible. Meanwhile, among 53 co-trimoxazole non-susceptible isolates, 44 (83 %) isolates displayed the Ile-100–Leu substitution. Meanwhile, 42 % these isolates (22/53) were Ile-100–Leu substitution isolates, along with Asp-92–Ala substitution isolates, followed by Ile-100–Leu substitution isolates, and Asp-92–Ala and Glu-94–Asp substitution isolates (32 %; 17/53) (Table 2). Most of the susceptible isolates (94 %; 15/16) did not show the Ile-100–Leu substitution. We found seven different variations with the Ile-100–Leu substitution (Table 2). We also discovered that the presence of the Ile-100–Leu substitution at DHFR resulted in higher resistance to co-trimoxazole (MIC ranged from 0.064/1.216–≥32/608 µg ml^−1^; median 12/228 µg ml^−1^) than isolates without the Ile-100–Leu substitution (MIC ranged from 0.064/1.216–≥32/608 µg ml^−1^; median 0.75/14.25 µg ml^−1^) regardless of the DHPS insertion.

**Table 2.** Substitution variation of the *folA* gene encoding the DHFR enzyme in *S. pneumoniae* isolates from the Indonesian population

Substitution at Codon 100	DHFR variation	No. (*n*=69)	Non-susceptible isolates (*n*=53)*, *n* (%)	Susceptible isolates (*n*=16)†, *n* (%)
Ile-100-Leu	Ile-100Leu	1	1 (2)	0
	Asp-92–Val, Ile-100–Leu	1	1 (2)	0
	Glu-94–Asp, Ile-100–Leu	1	1 (2)	0
	Asp-92–Ala, Glu-94–Asp, Ile-100–Leu	18	17 (32)	1 (6)
	Asp-92–Ala, Ile-100–Leu	22	22 (42)	0
	Asp-92–Ala, Glu-94–Asp, Lys-95–Asn, Ile-100–Leu	1	1 (2)	0
	Asp-92–Gly, Glu-94–Asp, Ile-100–Leu	1	1 (2)	0
None	Asp-92–Val	2	1 (2)	1 (6)
	Asp-92–Ala	18	7 (13)	11 (69)
	Wild-type	4	1 (2)	3 (19)

*Non-susceptible isolates with MIC range 0.75–32 µg ml^−1^.

†Susceptible isolates with MIC range <0,75 µg ml^−1^.

In this study, the combination of the Ile-100–Leu substitution at DHFR region and the 6 bp insertion was the most dominant combination (69 %; 25/36) among isolates having both *folA* and *folP* genes sequences, especially the combination of Asp-92–Ala, Glu-94–Asp, Ile-100–Leu and ST*RP*RPGSSYVEIE and the combination of Asp-92–Ala, Ile-100–Leu and ST*RP*RPGSSYVEIE (Table 3). Moreover, we also discovered that among isolates showing higher resistance (MIC ranged from 16/304–≥32/608 µg ml^−1^) to co-trimoxazole, the Ile-100–Leu substitution at the DHFR region and the 6 bp insertion were the most prevalent combination patterns. Moreover, we also discovered that among isolates showing higher resistance (MIC ranged from 16/304–≥32/608 µg ml^−1^) to co-trimoxazole, the Ile-100–Leu substitution at the DHFR region and the 6 bp insertion were the most prevalent combination patterns.

**Table 3.** The combination of DHFR and DHPS variation in *S. pneumoniae* isolates from the Indonesian population

DHFR variation	DHPS variation	*n* (%)
Asp-92–Ala	No mutation	5 (20)
Asp-92–Ala	ST*RP*RPGSSYVEIE	4 (16)
Asp-92–Ala, Glu-94–Asp, Ile-100–Leu	ST*RP*RPGSSYVEIE	8 (32)
Asp-92–Ala, Ile-100–Leu	ST*RP*RPGSSYVEIE	8 (32)

## Discussion

In this study, we observed an insertion mutation occurring in *folP* encoding dihydropteroate synthase (DHPS) and a substitution in *folA* encoding dihydrofolate reductase (DHFR), the enzymes involved in the folate biosynthesis pathway [14]. Insertions of 3 and 6 bp occurred in *folP*, with the 6 bp insertion showing more resistance compared to the 3 bp insertion. Furthermore, the 6 bp insertion was found to be the most prevalent insertion among co-trimoxazole non-susceptible isolates. This finding was consistent with previous studies that reported that these 3 and 6 bp insertions were also observed in the *folP* gene of co-trimoxazole non-susceptible isolates with the 6 bp insertion causing RP amino acid (ST*RP*RPGSSYVEIE) as a common mutation [10, 11]. In addition, we observed that the 6 bp insertion showed more resistance to co-trimoxazole compared to the 3 bp insertion and the wild-type. This finding was also reported in a study conducted in Tanzania, which showed that most isolates that were non-susceptible to co-trimoxazole carried multiple mutations in DHFR [15].

A substitution occurring in *folA* is also a factor causing resistance to co-trimoxazole. In this study, we discovered that some mutations occurred in the *folA* gene of co-trimoxazole non-susceptible isolates, with Asp-92–Ala and Ile-100–Leu substitutions being the most prevalent mutations (Table 2). This finding is consistent with a previous study which mentioned that substitutions in amino acid 92 (Asp-92–Ala) and 100 (Ile-100–Leu) were the most prevalent substitutions in co-trimoxazole non-susceptible isolates [10, 11, 15, 16]. Meanwhile, a study in Tanzania mentioned that Asp-92–Ala (53,1 %) and Ile-100–Leu (100 %) were also the dominant substitutions in the DHFR of trimethoprim/sulfamethoxazole non-susceptible isolates [15]. Further, we also detected the substitution at 94 (Glu-94–Asp) as the most common substitution after Asp-92–Ala and Ile-100–Leu, which is concordant with previously reported work [10, 15]. We also discovered some substitution combinations in DHFR (Table 2), but combination with Ile-100–Leu resulted in higher resistance against co-trimoxazole. The Ile-100–Leu was reported as an essential amino acid change for resistance development, while other mutations on DHFR did not show significant influence on resistance development. Other mutations without combination with Ile-100–Leu will not develop high resistance to co-trimoxazole [10, 11], but the substitution Asp-92–Ala without the substitution Ile-100-–Leu resulted in trimethoprim resistance [15]. We discovered that the Ile-100–Leu substitution in DHFR combined with the 6 bp insertion in DHPS shows higher resistance against co-trimoxazole (MIC ranged from 16/304–32/608 µg ml^−1^). The presence of these mutations reduced the affinity of trimethoprim in binding DHFR and sulfamethoxazole in binding DHPS, causing resistance to co-trimoxazole [10].

In summary, insertion in DHPS and substitution in DHFR lead to resistance against co-trimoxazole. The insertion of the 6 bp sequence into DHPS and the amino acid substitution Ile–100-Leu in DHFR seem to correlate with the development of resistance against co-trimoxazole. In addition, the combination of these mutations is associated with higher resistance to co-trimoxazole.
